# Pax7 haploinsufficiency impairs muscle stem cell function in Cre-recombinase mice and underscores the importance of appropriate controls

**DOI:** 10.1186/s13287-023-03506-1

**Published:** 2023-10-13

**Authors:** Despoina Mademtzoglou, Perla Geara, Philippos Mourikis, Frederic Relaix

**Affiliations:** 1 Univ Paris Est Creteil, INSERM, IMRB, F-94010 Creteil, France; 2 Ecole nationale vétérinaire d’Alfort, IMRB, F-94700 Maisons-Alfort, France; 3 EFS, IMRB, F-94010 Creteil, France; 4 AP-HP, Hopital Mondor, Service d’histologie, F-94010 Creteil, France

**Keywords:** Muscle stem cells, Satellite cells, Mouse molecular genetics, Stem cell research, Cre-lox, Pax7

## Abstract

Ever since its introduction as a genetic tool, the Cre-lox system has been widely used for molecular genetic studies in vivo in the context of health and disease, as it allows time- and cell-specific gene modifications. However, insertion of the Cre-recombinase cassette in the gene of interest can alter transcription, protein expression, or function, either directly, by modifying the landscape of the locus, or indirectly, due to the lack of genetic compensation or by indirect impairment of the non-targeted allele. This is sometimes the case when Cre-lox is used for muscle stem cell studies. Muscle stem cells are required for skeletal muscle growth, regeneration and to delay muscle disease progression, hence providing an attractive model for stem cell research. Since the transcription factor Pax7 is specifically expressed in all muscle stem cells, tamoxifen-inducible Cre cassettes (CreERT2) have been inserted into this locus by different groups to allow targeted gene recombination. Here we compare the two Pax7-CreERT2 mouse lines that are mainly used to evaluate muscle regeneration and development of pathological features upon deletion of specific factors or pathways. We applied diverse commonly used tamoxifen schemes of CreERT2 activation, and we analyzed muscle repair after cardiotoxin-induced injury. We show that consistently the Pax7-CreERT2 allele targeted into the Pax7 coding sequence (knock-in/knock-out allele) produces an inherent defect in regeneration, manifested as delayed post-injury repair and reduction in muscle stem cell numbers. In genetic ablation studies lacking proper controls, this inherent defect could be misinterpreted as being provoked by the deletion of the factor of interest. Instead, using an alternative Pax7-CreERT2 allele that maintains bi-allelic Pax7 expression or including appropriate controls can prevent misinterpretation of experimental data. The findings presented here can guide researchers establish appropriate experimental design for muscle stem cell genetic studies.

## Introduction

Skeletal muscle displays an incredible capacity to fully regenerate after severe injuries, thanks to a dedicated pool of muscle stem cells (MuSCs, also called satellite cells). Four groups showed independently that in the absence of MuSCs, skeletal muscle cannot recover from injuries [﻿^1^–^4^]. MuSCs have attracted much attention as a stem cell and regeneration model thanks to this extraordinary repair potential, but also because their malfunctioning could lead to (or fail to protect from) degenerative muscle disorders progression. To develop effective gene or cell therapies for these diseases, it is fundamental to elucidate the factors and pathways that ensure the correct function of the MuSCs. The Cre-lox genetic tool has been widely used for this purpose since it allows controlled spatio-temporal deletion of factors of interest, notably when using the estrogen receptor (ER) fusion alleles, CreER or CreERT2 [5], in which recombination is induced by the administration of exogenous estrogens (e.g., tamoxifen) that drive translocation of Cre recombinase to the nucleus.

*Pax7* regulatory elements have been commonly used as the driver of Cre recombinase for lineage tracing and genetic alterations in MuSCs. Several muscle labs generated Pax7-Cre, Pax7-CreER (or Pax7-CreER™) and Pax7-CreERT2 alleles [6]. The Pax7-CreERT2 generated by the Fan lab [7] is the first MuSC-specific CreERT2 to be produced and thus has been widely used for a long time. However, *CreERT2* is inserted off-frame in exon 1 of the *Pax7* locus rendering it null; indeed tamoxifen-treated *Pax7*^*CreERT2(Fan)/flox*^ mice had no *Pax7* transcript, Pax7 protein or Pax7 + cells in their muscles [7]. Thus, *CreERT2* insertion generates a *Pax7* heterozygous mouse, a parameter which has been disregarded in many studies (see below) but could affect muscle phenotype and ability to regenerate.

## Results and Discussion

**Fig. 1:**
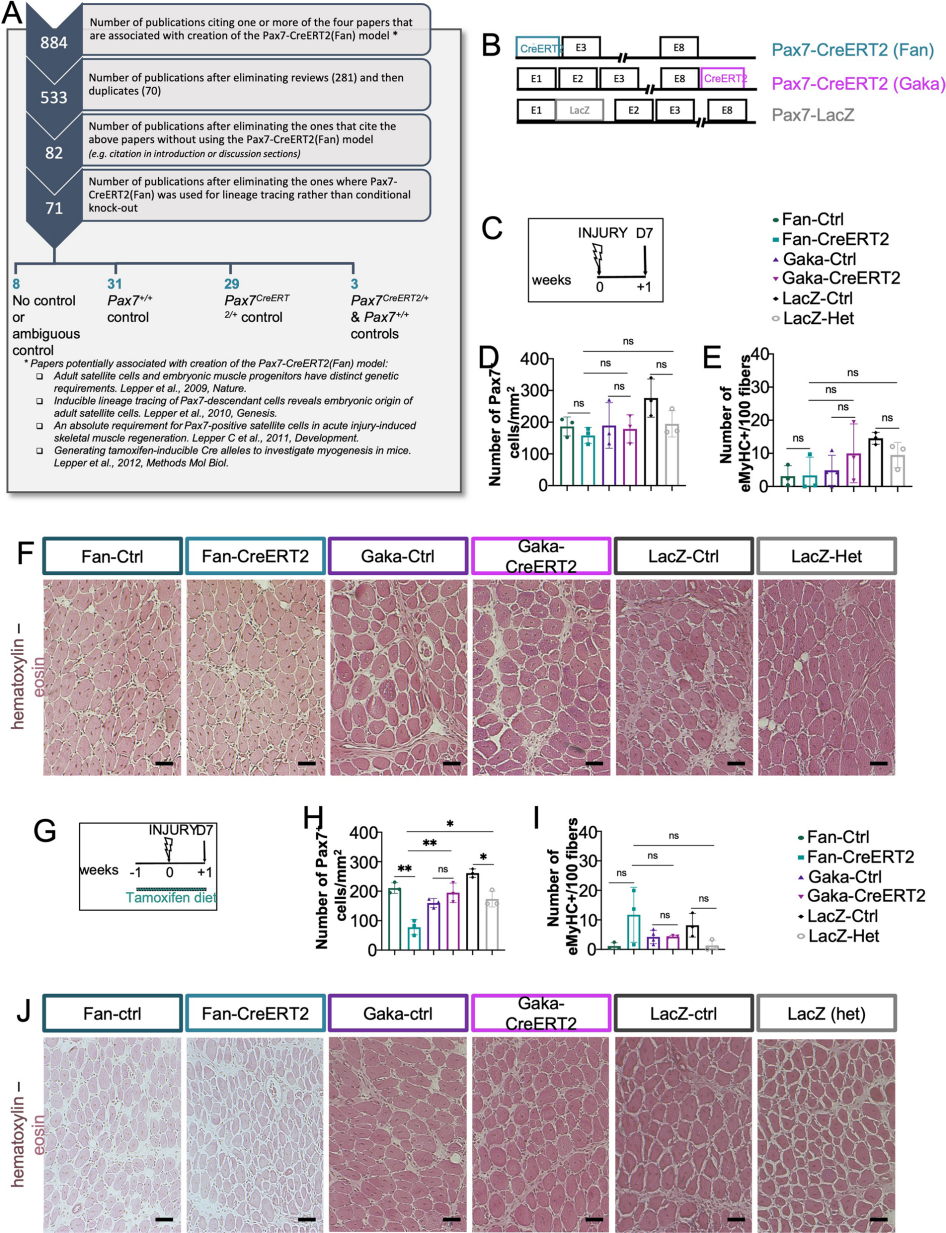
Assessing the regeneration of different *Pax7* alleles with no or limited tamoxifen. **A** Scheme of literature mining to identify studies using the *Pax7*^*CreERT2(Fan)*^ allele and used controls. Briefly, a list of all studies citing the *Pax7*^*CreERT2(Fan)*^ allele, and all associated studies was made. Reviews, duplicates, and studies that do not use the allele were removed. Finally, out of the remaining studies, controls were divided in 4 different groups (No control, WT control, Pax7^CreERT2^ control, or Pax7^CreERT2^ + WT controls). **B** Schematic representation of the different *Pax7* alleles used for this study. E1-E8 are exons 1 through 8. **C** Mice on regular diet, were injured with cardiotoxin (INJURY) and TAs were collected seven days post-injury (D7). **D**. Quantifications of the number of PAX7 + cells/mm^2^ from TA sections at seven days post-injury immunostained with PAX7 (MuSC marker), LAMININ (to outline the fibers), and DAPI (to counterstain nuclei). *n* = 3 per genotype. **E** Quantifications of the number of eMyHC + cells per 100 fibers from TA sections immunostained with eMyHC (regenerating fiber marker) and LAMININ (to outline all fibers). *n* = 3–4 per genotype. **F** Sections from TAs of injured mice were stained with hematoxylin (to highlight nuclei; purple) and eosin (to highlight the cytoplasm; pink). *n* = 3–4 per genotype. **G** Mice were put on tamoxifen diet for two weeks; TAs were injured with cardiotoxin (INJURY) following one week of the diet start and collected seven days following injury. **H** Sections from TAs of tamoxifen-treated, injured mice were immunostained with LAMININ and PAX7 and quantified for PAX7 + cells/mm^2^. *n* = 3–4 per genotype. **I** Quantifications of eMyHC + fibers per 100 fibers from TA sections immunostained with eMyHC and LAMININ following two weeks of tamoxifen diet. *n* = 3–4 per genotype. **J** Sections from TAs of tamoxifen-treated, injured mice were stained with hematoxylin (to highlight nuclei; purple) and eosin (to highlight the cytoplasm; pink). *n* = 3–4 per genotype. Independent Student’s t test was performed between the groups indicated by a horizontal bar in graphs and detailed in the Material and Methods section; ns, not statistically significant; *p < 0.05, **p < 0.01, and ***p < 0.001. Error bars indicate standard deviation. The experimental unit is single animals. Scale bar, 30 um

*Pax7*^*CreERT2(Fan)/*+^ mice are heterozygous for *Pax7* [7] and PAX genes often show heterozygous phenotypes in mouse models and human disease [8]. Given that Pax7 is important for proper MuSC function, it is imperative to compare *Pax7*^*CreERT2(Fan)*^-inducible mutants to *Pax7*^*CreERT2(Fan)/*+^ controls rather than *Pax7*^+*/*+^ controls. Thus, we first mined the literature for studies with the *Pax7*^*CreERT2(Fan)*^ allele and we reviewed the selected controls. We listed all papers that cited the original publication of the *Pax7*^*CreERT2(Fan)*^ allele [7], but also the three follow-up studies that could be mistaken for the original publication [1, 9, 10]. We then removed reviews, duplicates, and papers that cited the study without using the *Pax7*^*CreERT2(Fan)*^ mice. This left us with 71 papers in which a floxed gene was recombined by *Pax7*^*CreERT2(Fan)*^. Interestingly, more than half of those studies were using inappropriate controls (i.e., wild-type mice) and only 31 of them compared mutant phenotypes to control *Pax7*^*CreERT2(Fan)/*+^ allele (Fig. 1A).

Strikingly, most studies that used a Pax7-CreERT2 allele to delete a gene of interest performed muscle injury and analyzed the capacity of the conditional mutant muscle to regenerate, with the aim of revealing the gene(s) that drive MuSC function and could be used in a therapeutic context. Thus, an inherent regenerative problem of the Pax7-CreERT2 mouse line could be wrongly attributed as a phenotype caused by targeted deletion of the floxed gene. We, therefore, wanted to investigate the regeneration capacity of the two most used Pax7-CreERT2 alleles generated by the Fan and Kardon labs (hereafter *Pax7*^*CreERT2(Fan)*^ and *Pax7*^*CreERT2(Gaka)*^, respectively). In targeted alleles expressing CreERT2, *Cre* translocation to the nucleus is induced by tamoxifen; the effect of tamoxifen has been investigated in muscle and muscle diseases, but studies are lacking for MuSCs. In addition to untreated animals, we therefore analyzed cohorts under two (short induction) or five weeks of tamoxifen diet (achieving 95% recombination efficiency; data not shown). We chose tamoxifen diet over other administration methods driven by the: (i) need for less interventional animal treatments amid growing concerns on experimental animal welfare and increasingly strict regulations on experimental conditions, and (ii) lack of side effects on mouse physiology by tamoxifen diet, as opposed to intraperitoneal injections [11]. To evaluate the interactions between tamoxifen treatment, the Pax7-CreERT2 alleles, and muscle tissue repair, we injected cardiotoxin into the *Tibialis anterior* (TA) muscle, a standard model of muscle injury and regeneration of tamoxifen-treated mice. Regenerating muscles were collected seven days post-injection (D7), when they are expected to have newly formed myofibers and MuSCs renewal. The outcomes assessed post-regeneration were (i) general histology and muscle architecture (hematoxylin–eosin staining), (ii) numbers of MuSCs (Pax7 immunostaining), and iii) numbers of regenerated fibers (embryonic myosin heavy (eMyHC) immunostaining).

**Fig. 2:**
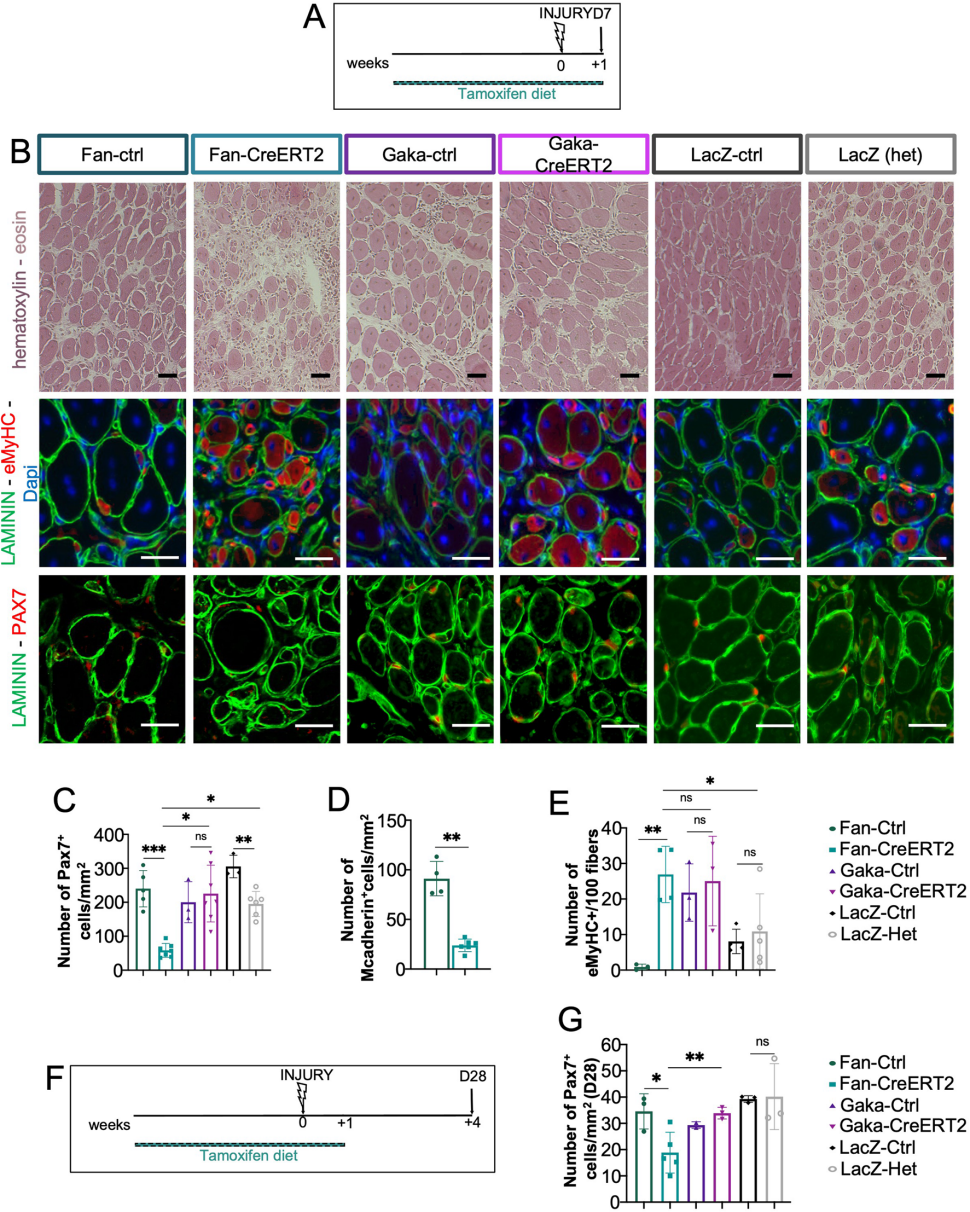
Assessing the regeneration of different *Pax7* alleles following prolonged tamoxifen administration. **A** Mice were treated with tamoxifen for five weeks total and injured with cardiotoxin (INJURY) following four weeks of treatment. TAs were then collected following seven days of injury. **B** Staining of sections from injured TAs for (i) upper panel: hematoxylin (to highlight nuclei; purple) and eosin (to highlight the cytoplasm; pink), (ii) middle panel: eMyHC (to mark newly formed regenerating myofibers; red), LAMININ (to outline myofibers; green), DAPI (to stain nuclei; blue) and (iii) lower panel: PAX7 (to mark MuSCs; red) with LAMININ (to outline myofibers; green) following seven days of injury in the five-week tamoxifen regimen. **C** Quantifications of PAX7 + MuSCs of (B). *n* = 3–7 per genotype. **D** Quantifications of mCadherin + cells/mm^2^ from injured TAs of *Pax7*^*CreERT2(Fan)/*+^ and controls from TA sections immunostained with mCadherin (MuSC marker) and LAMININ (to outline myofibers) collected at seven days post-injury following five weeks of tamoxifen treatment. *n* = 4–6 per genotype. **E** Quantifications of eMyHC + fibers per 100 fibers from TAs at day seven post-injury with five weeks of tamoxifen treatment. *n* = 3–5 per genotype. **F** Mice were treated with tamoxifen for five weeks total and injured with cardiotoxin (INJIURY) following four weeks of treatment. TAs were then collected following 28 days of injury. **G** Quantifications of the number of PAX7 + MuSCs from mice treated with five weeks of tamoxifen, injured following four weeks and TAs collected at 28 days post-injury. *n* = 2–5 per genotype. Independent Student’s t test was performed between the groups indicated by a horizontal bar in graphs and detailed in the Material and Methods section; ns, not statistically significant; *p < 0.05, **p < 0.01, and ***p < 0.001. Error bars indicate standard deviation. The experimental unit is single animals. Scale bar, 30 um

We analyzed the regeneration capacity of the *Pax7*^*CreERT2(Fan)/*+^ and *Pax7*^*CreERT2(Gaka)/*+^ mice, which respectively contain a mutant or an intact *Pax7* allele, because the Cre was inserted in Exon 1 or as a bicistronic IRES transcript in the 3’ UTR, respectively (Fig. 1B). Of note, even though *Pax7* expression has not been properly quantified in *Pax7*^*CreERT2(Gaka)*^, analysis of muscles for which Pax7 function is crucial (i.e., at early postnatal and post-regeneration stages) showed that Pax7 function is not compromised in tamoxifen-treated *Pax7*^*CreERT2(Gaka)/CreERT2(Gaka)*^ compared to controls [3]. As an internal control of Pax7 loss, our analysis includes the *Pax7*^*LacZ*^ mouse that also harbors a *Pax7* null allele (Fig. 1B) and displays Pax7 mutant phenotypes [12],specifically, β-galactosidase and neomycin were inserted in the first exon of the paired box of Pax7, leading to no detectable *Pax7* RNA in *Pax7*^*LacZ/LacZ*^ mice [12]. Because *Pax7*^*CreERT2(Fan)*+^, *Pax7*^*CreERT2(Gaka)/*+^, and *Pax7*^*LacZ/*+^ mice come from slightly different genetic backgrounds, we used *Pax7*^+*/*+^ littermates as controls for each mouse line. Histological analysis of regenerating muscles did not reveal gross morphological differences in any line (Fig. 1C–J). In the absence of tamoxifen treatment, there was no significant difference in the number of PAX7 + cells (i.e., MuSCs) and eMyHC + myofibers (i.e., newly formed, regenerated fibers; Fig. 1C–E). However, two weeks of tamoxifen treatment decreased MuSC numbers in *Pax7*^*CreERT2(Fan)/*+^ and *Pax7*^*LacZ/*+^ but not *Pax7*^*CreERT2(Gaka)/*+^ muscles, suggesting a combinatorial regeneration delay due to Pax7 haploinsufficiency in the presence of tamoxifen (Fig. 1G–I). This phenotype was even more evident with five weeks of tamoxifen treatment (Fig. 2A–E). In this tamoxifen regimen, MuSCs were reduced in *Pax7*^*CreERT2(Fan)/*+^ and *Pax7*^*LacZ/*+^ mice, but not *Pax7*^*CreERT2(Gaka)/*+^, as revealed by PAX7 + counts in all models and confirmed by mCadherin + cells in *Pax7*^*CreERT2(Fan)/*+^ (Fig. 2B–E). Furthermore, the regeneration delay was also evident in the *Pax7*^*CreERT2(Fan)/*+^ by the increased numbers of eMyHC fibers compared to its control (Fig. 2B, E). In addition, at five weeks of tamoxifen diet, hematoxylin–eosin staining revealed considerable regions that were not repaired in *Pax7*^*CreERT2(Fan)/*+^ samples, as opposed to their controls or other lines (Fig. 2B). Given the severe D7 regenerative phenotype in the five-week tamoxifen regimen, we then evaluated the injured muscles 28 days post-injury, when the damaged muscles are expected to display a complete recovery. At this late stage, *Pax7*^*CreERT2(Fan)/*+^ TAs still showed a reduced number of PAX7 + MuSCs compared to their controls, while we did not observe significant differences in *Pax7*^*LacZ/*+^ and *Pax7*^*CreERT2(Gaka)/*+^ TAs compared to their respective controls (Fig. 2F–G).

In conclusion, we report that *Pax7*^+/-^ mice (i.e., *Pax7*^*CreERT2(Fan)/*+^ or *Pax7*^*LacZ/*+^) present with a defect in regeneration following prolonged tamoxifen treatment. *Pax7*^*CreERT2*(Gaka)*/*+^ mice do not show regeneration defects in our (Figs. 1 and 2) or previous studies [3], supporting the idea that the defects in *Pax7*^*CreERT2(Fan)/*+^ or *Pax7*^*LacZ/*+^ are due to Pax7 heterozygosity. Our study therefore highlights the critical importance of selecting the correct genetic tools with appropriate controls to study biological mechanisms and diseases. This is recognized by the lab that created the *Pax7*^*CreERT2(Fan)/*+^ mice and that consistently compares conditional knockouts to Pax7-CreERT controls. However, attention should be drawn since the main commercial provider of these mice recommends wild-type littermates as controls (jax.org/strain/012476; Accessed 23 Sept 2023). Moreover, our analysis revealed that approximately half of the published studies using this allele have not included the appropriate controls. Pax7 null mice show dysgenesis of cephalic neural crest derivatives and have limited to no survival to adulthood (genetic background-dependent), while the few that survive have thinner muscles that can barely regenerate [12–15]. Their MuSCs display enhanced cell death and differentiation [8, 10], yet it is unclear whether the reduction that we observed in regenerating Pax7 heterozygote animals –especially *Pax7*^*CreERT2(Fan)/*+^ – is a result of cell death, precocious activation and inappropriate differentiation, or impaired proliferation, ineffective self-renewal, or even additive effects between inherent *Pax7* heterozygous phenotypes and either Cre [16] or tamoxifen treatment, the latter also having been associated with inguinal hernias [17]. Regardless of the mechanism, *Pax7* heterozygosity results in defective muscle regeneration in the presence of tamoxifen, and, thus, our study highlights the importance of carefully designing mechanistic studies of stem cells with appropriate Cre alleles and respective controls.

## Material and methods

### Study analysis

In June 2022 we mined PubMed for all papers that cited one or more of the four publications that describe the *Pax7*^*CreERT2(Fan)*^ allele by the group that generated it [1, 7, 9, 10]. We then removed reviews and duplicates. The remaining papers were categorized as shown in Table 1 and as summarized in Fig. 1A.

**Table 1:** Publications citing one or more of the four publications that describe the *Pax7*^*CreERT2(Fan)*^ allele by the group that generated ﻿it

Category	PMID numbers of publications
Publications in which the *Pax7*^*CreERT2(Fan)*^ allele is not used (e.g., the Lepper et al. or Lepper & Fan publications are cited in the introduction or discussion sections)	20118923, 23728463, 26328519, 26999737, 30097922, 32966776, 33222146, 33895984, 34481290, 19835858, 19920078, 20144785, 20175910, 20181926, 20442248, 20483998, 20605921, 20841498, 20887952, 21270051, 21374762, 21464233, 21478154, 21609392, 21645366, 21828091, 21828094, 21931825, 21946848, 21988915, 21989910, 22037676, 22045613, 22076929, 22096526, 22130829, 22130844, 22159413, 22203957, 22216009, 22266944, 22302939, 22371254, 22385659, 22439962, 22470452, 22490927, 22493066, 22523551, 22537489, 22546853, 22564549, 22609161, 22662253, 22692546, 22730231, 22848431, 22863532, 22887880, 22895262, 22905258, 22945933, 23021218, 23038772, 23046558, 23070814, 23149873, 23154418, 23166781, 23247248, 23336007, 23349935, 23395168, 23464362, 23485971, 23499423, 23505517, 23517218, 23527126, 23554995, 23569214, 23619364, 23631552, 23639729, 23671652, 23700507, 23743995, 23774280, 23781029, 23791730, 23818608, 23840772, 23911934, 23922277, 23954233, 24055173, 24067916, 24092696, 24150075, 24170859, 24336075, 24375410, 24376025, 24439384, 24524008, 24531379, 24587351, 24647690, 24678443, 24687582, 24703692, 24706792, 24709624, 24715455, 24743741, 24753152, 24844572, 24844636, 24883236, 24898588, 24906148, 24940559, 25068087, 25103976, 25157816, 25194572, 25197048, 25241745, 25281303, 25376879, 25410765, 25414242, 25487404, 25501907, 25520397, 25543153, 25578880, 25671422, 25706128, 25750654, 25848030, 25866834, 25869487, 25878030, 25960061, 25971691, 26028225, 26042028, 26090310, 26151913, 26157009, 26178867, 26230763, 26278933, 26304770, 26312504, 26317052, 26333785, 26379543, 26384382, 26413555, 26447161, 26457071, 26491708, 26494623, 26503169, 26510925, 26526875, 26579218, 26607845, 26625958, 26649785, 26666572, 26679610, 26721734, 26739770, 26743565, 26784637, 26796754, 26807982, 26839378, 26876562, 26904948, 26923583, 26949601, 26949720, 27138650, 27144473, 27144531, 27159388, 27193340, 27239402, 27239431, 27303309, 27343167, 27349181, 27350317, 27446912, 27452271, 27452471, 27531927, 27583644, 27589388, 27628322, 27644105, 27690836, 27699827, 27725085, 27765651, 27768064, 27826411, 27849571, 27854222, 27922824, 27923399, 27927611, 27932789, 27965322, 28025671, 28096434, 28169594, 28173861, 28174253, 28279710, 28287512, 28344332, 28369879, 28400501, 28417015, 28498863, 28599679, 28605361, 28615691, 28693603, 28698658, 28701357, 28714082, 28721121, 28735515, 28736900, 28833130, 28855574, 28874575, 28885620, 28887016, 28935952, 28939843, 28943254, 29017757, 29030619, 29031720, 29040321, 29041952, 29073096, 29075589, 29115986, 29116170, 29127611, 29231808, 29291980, 29293645, 29302341, 29315567, 29324825, 29329332, 29337118, 29337929, 29444710, 29463296, 29476012, 29523096, 29523805, 29540680, 29557384, 29581096, 29641992, 29674978, 29681515, 29694232, 29706500, 29731431, 29733324, 29800621, 29849047, 29881353, 29892004, 29896117, 29906337, 29973273, 29991029, 30016973, 30045918, 30054310, 30062184, 30081710, 30116776, 30139374, 30139390, 30177888, 30202063, 30300394, 30305290, 30367407, 30368256, 30370532, 30382921, 30404653, 30414844, 30416048, 30501636, 30510196, 30524716, 30526691, 30537304, 30548460, 30577454, 30581652, 30648479, 30683662, 30733760, 30753815, 30862660, 30867438, 30872480, 30916493, 30949196, 30972959, 30982893, 31006621, 31006622, 31021652, 31091443, 31093982, 31160583, 31187089, 31191361, 31242425, 31268301, 31325224, 31366088, 31474563, 31513447, 31518419, 31578935, 31661460, 31699935, 31710288, 31719873, 31724771, 31809738, 31852888, 31909353, 31915055, 31931473, 31947603, 31991048, 32011372, 32019766, 32025579, 32116540, 32123818, 32130242, 32204424, 32273524, 32283390, 32361680, 32390569, 32391357, 32393776, 32413092, 32414934, 32478048, 32523512, 32541004, 32552916, 32613162, 32678274, 32708412, 32762770, 32763161, 32851730, 32905522, 32943091, 32980698, 33036659, 33047485, 33081771, 33083017, 33102913, 33125370, 33126429, 33170806, 33244887, 33372732, 33374379, 33377052, 33473298, 33551852, 33602287, 33641201, 33664851, 33664851, 33732701, 33800595, 33805924, 33848270, 33864373, 33916485, 33941806, 34059674, 34074531, 34083673, 34119711, 34218527, 34277616, 34319911, 34351905, 34383202, 34388363, 34413292, 34485607, 34535684, 34650976, 34676210, 34739848, 35326452, 35463471, 34334511, 34481290, 34560818, 34650038, 34676213, 34704386, 34750174, 34784301, 34940613, 35021050, 35023852, 35177647, 35323108, 35356683, 35380608, 35395625, 35574443, 34944549, 22562803, 32364045, 22290419, 23708921, 30852688, 31734897 (Total: 451 publications)
Publications in which the *Pax7*^*CreERT2(Fan)*^ allele was used for lineage tracing rather than conditional knock-out	33131406, 20641127, 22869749, 23760954, 25133429, 26686466, 27918914, 28218909, 29091764, 29643389, 31545169 (Total: 11 publications)
Publications in which the *Pax7*^*CreERT2(Fan)*^ allele was used for conditional knock-out and there is no control or ambiguous control	27548913, 25761890, 32433961, 24239359, 24276242, 25503558, 25636474, 29156133 (also used for lineage tracing) (Total: 8 publications)
Publications in which the *Pax7*^*CreERT2(Fan)*^ allele was used for conditional knock-out and the control is *Pax7*^+*/*+^	22279050, 23079662, 23582327, 23933088, 24065826, 24591619, 25069613, 25205686, 26304725, 26453297, 26603188, 26648529, 27594590, 27743478, 27880908, 28094257, 28232488, 28694257, 28790171, 29371665, 29581287, 29882512, 30283903, 31012848, 31277245, 31693883, 32310830, 32822588, 33328166, 34108202, 35342888 (Total: 31 publications)
Publications in which the *Pax7*^*CreERT2(Fan)*^ allele was used for conditional knock-out and the control is *Pax7*^*CreERT2/*+^	19554048, 21828092, 24084740 (with the exception of supplementary Fig. 12, in which the control is *Pax7*^+*/*+^), 24260586 , 24906372, 25085416, 25973951, 26999603, 27239264, 27346341, 27376575, 27376579, 27825107, 28186492, 28332979, 28463680, 28515121, 29395054, 30279563, 30979776, 31534153, 32021964, 32103583, 32240622, 32275813, 34093712, 34350830, 35228746, 35243267 (Total: 29 publications)
Publications in which the *Pax7*^*CreERT2(Fan)*^ allele was used for conditional knock-out and there are two controls: *Pax7*^*CreERT2/*+^ and *Pax7*^+*/*+^	29269426, 29472596, 30284969 (Total: 3 publications)

### Mice

The following mouse (*Mus musculus*) lines were provided by the corresponding laboratories as described: *Pax7*^*CreERT2(Fan)*^ [7], *Pax7*^+*LacZ*^ [12], *Pax7*^*CreERT2(Gaka)*^ (G. Kardon; Jackson Laboratories, stock 017763; [3]. Animals were handled as per French and European Union guidelines, and protocols were approved by the French Ministry of Higher Education, Research and Innovation following favorable opinion of the ethics committee 16/Anses-ENVA-UPEC (Project No: 20–027 #27431). The same ethics committee approved our study design in terms of animal numbers taking into account statistical power and the “Reduction” aspect of the 3R principle. We analyzed a minimum of three animals per genotype per line. Adult (8–12 week old) animals were imported weekly from the breeding facility at Orleans (France) to the experimental facility at Creteil (France) and they were given minimum seven days to adjust to the new conditions. A priori exclusion criteria were (i) death before inclusion to an experimental group (details in Table 2) and ii) the rare event that the TA was not fully injected, or it broke before cryosectioning and analysis (details in Table 2). In the above cases, a new control-mutant set was imported into the experimental facility; thus, the compared groups occasionally have *n* > 3. A total of 76 animals were analyzed in this study.

**Table 2:** List of animals excluded from analysis

	No tamoxifen, analysis at D7	Five weeks tamoxifen, analysis at D7	Five weeks tamoxifen, analysis at D28
Excluded animals	Gaka-CreERT2 (1 mouse) LacZ-Ctrl (1 mouse)	Fan-CreERT2 (1 mouse) Gaka-Ctrl (1 mouse) LacZ-Ctrl (3 mice)	Gaka-Ctrl (2 mice) Gaka-CreERT2 (1 mouse)

In both facilities mice are kept in rooms, cages, and enrichment environments that are up to the highest EU and French standards and regulations; both facilities have been audited and certified and further information on housing and husbandry can be requested to the facility heads. In the context of the tamoxifen and injury experimental procedures of this study (details below), the following methods were established and approved by the ethics committee to minimize or deplete pain and suffering: (i) mouse handling only by authorized researchers that have passed the two-week initial animal experimentation training and relevant continuous education required in France, (ii) mouse follow-up by experienced animal caretakers, including weekly checks; as a general practice, when signs of suffering are presented (e.g., distress, muscular atrophy, cachexia, respiratory difficulties, ulcers, etc.), the animals are euthanized, (iii) placement of hydrogel and wet food on cage floor, in case of reduced mobility after the muscle injury procedure [in that case the animal is checked more often for the signs listed in (ii)], (iv) acclimatization of at least seven days after importation to the experimental facility, (v) animal caretaker training for housing amelioration, for example cage enrichment with cardboard tunnels or plastic shelters.

### Tamoxifen treatment and injury

Adult (8–12 weeks of age) male mice were placed on tamoxifen diet (Envigo; TD.55125.I) for two (Fig. 1G-J) or five weeks (F﻿ig. 2), as indicated in the figure, and were observed regularly for signs of poor tolerance (weight loss, suffering, apathy, dehydration, etc.). For muscle injuries, mice were anesthetized with 3% isoflurane, and injured by intramuscular injection of 50 uL cardiotoxin at 10 umol (Latoxan; L8102). Specifically, in a dedicated room of the experimental animal facility, mice were put in dedicated transparent plastic cages that had isoflurane and oxygen flow until anesthetized; once anesthetized then they were transferred on warming plates to be injected under continuing inhalation anesthesia. Once injected they were transferred back to their cage and observed until waking up, followed by daily observation for signs of poor tolerance (weight loss, suffering, apathy, dehydration, etc.). At seven (Figs. 1, 2﻿) or 28 days post-injury (Fig. 2), mice were sacrificed by cervical dislocation and their TA muscles were collected for analysis. We observed no adverse events that could be attributed to the procedures in which the animals were subjected.

### Immunostaining

Muscles were frozen fresh in liquid nitrogen-cooled isopentane and sectioned at 8um with a Leica cryostat. Sections were first fixed with 4% paraformaldehyde for 10 min and washed 3 times with PBS. The sections were then permeabilized with 0.5% Triton-X (Sigma) for 8 min, washed with PBS, then blocked with 5% BSA (Jackson Laboratories 001–000-162) for 1 h at room temperature. Sections were then incubated with the primary antibodies overnight at 4 ^o^C. The next day, samples were washed with PBS, incubated with the secondary antibodies for 1 h at room temperature, washed with PBS, and mounted.

For PAX7 staining, samples were permeabilized with cold methanol for 6 min instead of Triton-X, washed with PBS, then boiled in citric acid (10 mM, pH6) for 3 min, then let cool down at RT for 30 min. Sections were then washed with PBS, blocked with 5% BSA and incubated with Fab (Interchim; 115–007-003) for 30 min at RT. The staining was then completed as described above.

All antibodies are listed in Table 3.

**Table 3:** List of antibodies

Antibodies	References	Dilution
IgG1 mouse monoclonal anti-Pax7	Santa Cruz sc-81648	IF 1/200
Rabbit polyclonal anti-Laminin	Sigma L9393	IF 1/500
IgG1 mouse monoclonal eMyHC	Santa Cruz sc-53091	IF 1/200
Mouse monoclonal mCadherin	Nanotools MCAD-12G4	IF 1/100
Goat anti-mouse IgG1, Alexa Fluor 555 conjugated	Molecular Probes; A-21127	IF 1/1000
Goat anti-rabbit IgG, Alexa Fluor 488 conjugated	Molecular Probes A-11008	IF 1/1000

### Histology

Muscles were frozen fresh in liquid nitrogen-cooled isopentane and sectioned at 8 mm with a Leica cryostat. Sections were fixed with 4% paraformaldehyde for 20 min at room temperature and washed twice with PBS (10 min per wash). Nuclei were stained with hematoxylin (Sigma) for 25 min and cytoplasms were counterstained with eosin (Sigma) for 40 s. The sections were then dehydrated with 30 s passages through increasing concentrations of ethanol (30%, 50%, 70%, 85%, 95%), followed by 15 min in absolute ethanol.

### Statistical analyses

For comparison between two groups, unpaired Student’s t test was performed to calculate *p* values and to determine statistically significant differences using Graphpad’s Prism software (*p < 0.05; **p < 0.01; ***p < 0.001; ****p < 0.0001). The experimental unit was single animals (data per animal are presented in Figs. 1, 2; exact values can be provided upon request). We primarily compared the control and mutant groups of each line (*Pax7*^*CreERT2(Fan)*^: Ctrl VS CreERT2, *Pax7*^*CreERT2(Gaka)*^: Ctrl VS CreERT2, *Pax7*^*LacZ*^: Ctrl VS het). When indicated in the figure, we also compared mutants of different lines (Fan-CreERT2 VS Gaka-CreERT2 or Fan-CreERT2 vs LacZ-het).

## Data Availability

All data generated or analyzed during this study are included in this published article.

## Abbreviations

eMyHC: Embryonic myosin heavy chain
ER: Estrogen receptor
MuSC: Muscle stem cells
TA: *Tibialis anterior*
